# Modeling cancer’s ecological and evolutionary dynamics

**DOI:** 10.1007/s12032-023-01968-0

**Published:** 2023-02-28

**Authors:** Anuraag Bukkuri, Kenneth J. Pienta, Ian Hockett, Robert H. Austin, Emma U. Hammarlund, Sarah R. Amend, Joel S. Brown

**Affiliations:** 1 Cancer Biology and Evolution Program and Department of Integrated Mathematical Oncology, Moffitt Cancer Center, Tampa, USA; 2 The Brady Urological Institute, Johns Hopkins School of Medicine, Baltimore, USA; 3 Department of Physics, Princeton University, Princeton, USA; 4 Tissue Development and Evolution Research Group, Department of Laboratory Medicine, Lund University, Lund, Sweden

**Keywords:** Eco-evolutionary dynamics, Mathematical modeling, Evolutionary game theory, Resistance, Cancer evolution

## Abstract

In this didactic paper, we present a theoretical modeling framework, called the *G*-function, that integrates both the ecology and evolution of cancer to understand oncogenesis. The *G*-function has been used in evolutionary ecology, but has not been widely applied to problems in cancer. Here, we build the *G*-function framework from fundamental Darwinian principles and discuss how cancer can be seen through the lens of ecology, evolution, and game theory. We begin with a simple model of cancer growth and add on components of cancer cell competition and drug resistance. To aid in exploration of eco-evolutionary modeling with this approach, we also present a user-friendly software tool. By the end of this paper, we hope that readers will be able to construct basic *G* function models and grasp the usefulness of the framework to understand the games cancer plays in a biologically mechanistic fashion.

## Introduction

Traditionally, scientists have taken a gene-centric view towards cancer, focusing on the genetic and molecular mechanisms underlying oncogenesis without consideration of the broader ecological and evolutionary factors at play. Despite the billions of dollars poured into cancer research over the last several decades, progress has been disappointing. In recent years, however, there has been a growing appreciation among both theorists and empiricists for cancer as an ecological and evolutionary process [35, 58, 100]. This view has led to a better understanding of the initiation and spread of cancer [2, 12, 45, 95], innovations in evolutionarily informed therapies [19, 20, 47, 105], and a reframing of the way we think about cancer [5, 17, 51, 76, 83, 91]. In this paper, we introduce an evolutionary game theoretic (EGT) approach called *G* functions that allow us to mathematically formalize notions of ecology and evolution in cancer. Due to the pedagogic nature of the exposition, we begin from first principles and guide the reader through how to construct basic models of cancer dynamics. The goal is not to elucidate previously unknown aspects of oncogenesis, but rather to show the reader how to construct *G* function models of cancer and convince them of its usefulness to understand cancer.

The *G*-function mathematically captures the ecological and evolutionary dynamics that impact the fitness of a species, as reflected in its per capita growth rate [18, 103]. This modeling framework, inspired by traditional quantitative genetics approaches, has traditionally been used to investigate problems in evolutionary ecology such as predator–prey coevolution [15] or consumer-resource games [87]. Recently, it has been adapted to explore problems in cancer [19–21, 27, 28, 86]. The hallmarks of cancer provide a useful perspective to form the basis of a fitness generating *G*-function framework for cancer [52]. The hallmarks of resisting programmed cell death, evading anti-growth signaling, and reproductive immortality state the necessary conditions for any organism to be defined as a species and a unit of natural selection. Specifically, these features emphasize that the cancer cell lineage is a distinct species from its multicellular host. Other hallmarks– deregulated cell metabolism, genetic instability, and sustained growth signaling–describe early events in a cancer cell’s adaptation to its environment, in which it ceases to serve its host and transitions towards exploiting it. Finally, the hallmarks inducing new blood flow, tumor promoting inflammation, and avoiding immune responses describe the kinds of adaptations that any organism evolves via natural selection in response to resource limitations, competition, and threats from hazards [91]. Mathematically, these characteristics become the evolving heritable traits (strategies) built into a *G*-function that influence its population dynamics via replication (proliferation) and deaths.

*G*-function models allow for the consideration of heritable strategies. Though not explicitly defining underlying genetics and epigenetics that permit heritable phenotypic variation, *G*-function models instead focus on the phenotypes that directly influence an cell’s fitness. The cancer *G*-function models presented here and on the publicly available website (https://lively wave-033dd4510.azurestaticapps.net/) utilize deterministic ordinary differential equations to capture cancer cells’ ecology and link their divisions and deaths to their heritable strategies and circumstances. This permits generality. Features such as space (through agent-based models, partial differential equations, or graph-based methods) [1, 10, 14, 25, 56, 63, 75, 96, 106, 108] and stochasticity (demographic and environmental) [30–33], though not discussed here, can be developed as extensions or analogs. *G*-function models are also quite similar to those from the field of adaptive dynamics [57, 66, 68, 69], though the latter has not been widely applied to cancer [3].

This *G*-function framework for cancer focuses on the perspective of a single cancer cell (i.e., after cancer initiation has occurred) and its subsequent growth rate within a population of cancer cells. A population of cancer cells is under selection to maximize fitness (proliferation rate minus death rate) given its circumstances. These circumstances include its physical environment, resource availability, presence of toxins and metabolites, normal cells (e.g., fibroblasts and immune cells), other cancer cells, and the strategies of these other cancer cells. All of these become potential components of a mathematical model. In particular, the model becomes game theoretic when the fitness of a cancer cell depends on its strategy, the presence of neighboring cancer cells, and the strategies of those cancer cells. Not only is cancer its own species, it is also an evolutionary game, and the *G*-function framework was developed to specifically consider the kinds of evolutionary games manifested by cancer. Like the approach proposed here, most mathematical models of cancer population and evolutionary dynamics have roots in models of population ecology and consider the cancer as its own “species” distinct from the whole organism [44, 59, 74, 89].

In this paper, we discuss how cancer is an evolutionary game and show how *G*-functions are a useful tool to understand the games cancer plays, from cell-cell competition to the evolution of therapeutic resistance. We begin by introducing the *G*-function framework, starting with Darwin’s theory of natural selection. We then construct equations for ecological (population) and evolutionary (strategy) dynamics. Next, we construct and simulate *G*-functions in the context of cancer, starting with a core cancer growth model and adding on cancer cell competition. We end the paper with an example of the emergence of therapeutic resistance, showing insights that can be gleaned from using the *G*-function framework. In this process, we introduce a software tool for modeling *G*-functions that allows researchers to extensively explore the models presented in this paper without requiring a detailed understanding of the mathematics underlying our models. All model simulations in this paper were directly generated from the tool itself. The unique power of *G*-functions to simultaneously capture ecological and evolutionary dynamics in a biologically mechanistic way makes it a powerful tool to understand cancer. We expect in years to come that this approach will be adopted by mathematical modelers, experimental cancer biologists, and clinical oncologists to aid in hypothesis generation, testing, and clinical decision-making.

## Methods

The cancer *G*-function is based on the fundamental tenets of Darwin’s theory of natural selection. The three core principles are: (1) there must be heritable variation such that like begets like with alterations, (2) there must be a struggle for existence that prevents populations from growing without bound, (3) heritable variation must influence this struggle-some phenotypes (strategies) beat others. The first assumption of heritable variation is captured by each clone in the population having a heritable strategy, $u_i$. This heritable strategy belongs to an evolutionary strategy set, *U*, which delineates the set of plausible values this strategy can take, providing biologically realistic limits to evolution. These strategies could represent transporter expression levels or resistance to a drug via gene expression, for example, and could be bounded by the maximum number of transporters a cell can express or the complete resistance to a drug (i.e., the drug does not induce any death in the cell population). These bounds prevent unreasonable evolutionary outcomes such as evolving a near-infinite number of drug antiporters. The struggle for existence is captured through the fitness generating function, $G(v, {\textbf{u}}, {\textbf{x}})$, which represents how a clone’s per capita growth rate (the rate at which the population size changes per cell in the population) depends on the strategies (**u**) and population densities (**x**) of all clones in the population. The impact of heritable variation is captured by the dependence of the fitness generating function on the clone’s strategy, *v*.

To avoid confusion, it’s worth delineating the difference between evolutionarily identical groups, clones, and cells. Cells with the same strategy set (*U*) and those that experience the same consequences of possessing those strategies are part of the same evolutionarily identical group. In other words, cells with the same *G* function can be grouped into an evolutionarily identical group, obviating the need to construct a separate *G* function for each cell. Clones represent cells that are not only evolutionarily identical, but also possess the same strategy. Typically, populations are composed of multiple clones (otherwise known as morphs). However, in the case of a monomorphic population, describing the clone and describing the population are analogous. For expositional purposes, we will often use clones, morphs, and cells interchangeably.

In some biological phenomena, there is a clear time-scale separation between ecological (population) and evolutionary (strategy) dynamics: often, the ecological time scale is much faster than the evolutionary time scale. In these contexts, it may be acceptable to simply model ecological dynamics and ignore the evolutionary underpinnings. However, in scenarios such as the evolution of drug resistance [16], the evolutionary trajectories of cells play a key role in the persistence of the population. To formulate a complete mathematical theory of evolution by natural selection, both ecological and evolutionary dynamics must be considered. The power of *G* functions comes from their ability to simultaneously consider both these components. Here, we build simple ordinary differential equations for each. First, we consider ecological dynamics. Since the fitness generating function *G* was defined as the *per capita growth rate*, to determine the growth of the entire clone, we multiply this rate by the number of cells in the clone. Doing this gives us the following equation that governs the change in each clone’s population size over time:

1 $$\begin{aligned} \frac{\mathrm{{d}}x_i}{\mathrm{{d}}t} = x_iG(v, {\textbf{u}}, {\textbf{x}})|_{v=u_i}. \end{aligned}$$

**Fig. 1:**
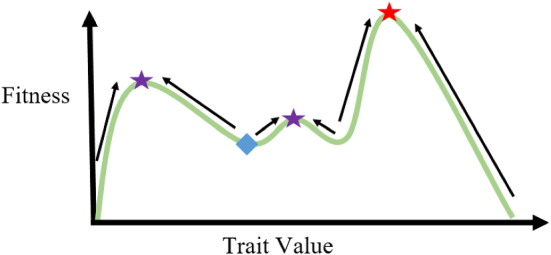
Adapted from [18]. An adaptive landscape that plots the fitness (*y*-axis) of an cell with a given trait value (*x*-axis). Though depicted here as a single snapshot in time, in reality, adaptive landscapes dynamically change in response to trait frequencies and population sizes

Next, we construct an equation which describes evolutionary dynamics: how the population’s trait value changes over time. To do this, we must consider both in which direction and how fast the trait evolves. The direction is governed by how changes in strategy affect fitness. This is given by the (local) gradient of the *G* function, which captures how a small increase in the trait value impacts the per capita growth rate of a clone. The trait will evolve in the direction that increases fitness, i.e., if an increase in drug antiporters increases the per capita growth rate of the clone, it will be evolutionarily favored. The rate at which the trait evolves depends on the slope of the adaptive landscape–the steeper the slope, the faster the trait will evolve. In other words, if the selection pressure induced by a drug is extreme, cells will evolve resistance more quickly. The rate of evolution also depends on the trait’s evolvability, which captures the trait’s ability to respond to natural selection. Evolvability can be influenced by several factors including mutation rate and the underlying genetic architecture. The more evolvable the trait, the faster the rate of evolution. These ideas can be formalized in the following equation:

2 $$\begin{aligned} \frac{\mathrm{{d}}u_i}{\mathrm{{d}}t} = k\frac{\mathrm{{d}}G}{\mathrm{{d}}v}\Big |_{v=u_i}, \end{aligned}$$

where *k* is a measure of the trait’s evolvability and $\frac{\mathrm{{d}}G}{\mathrm{{d}}v}$ is the gradient of the fitness generating function. Strategy dynamics are often visualized on an *adaptive landscape*, such as the one shown in Fig. 1. This landscape is produced by computing the fitness (per capita growth rate) at each time step as a function of the focal cell’s strategy, *v*. As the strategies and population densities of clones in the population change, so too does the adaptive landscape.

Assume the blue diamond in Fig. 1 represents the current location of the cells’ trait. At each time step, cells produce progeny with trait values in a local neighborhood of their own trait value. The cells with traits that confer a higher fitness will persist in the population, while those with traits that endow a lower fitness will go extinct. In this process, as a mean field approximation, the clone scales the landscape to reach a local fitness maximizing peak, as can be seen in the figure by the arrows. At each time step, the trait changes based on the slope of the adaptive landscape ($\frac{\mathrm{{d}}G}{\mathrm{{d}}v}$) and the “step size” (*k*), with local and favorable alternatives being preferred at each step. Although *k* may be variable [20, 21], changing as a function of how stressed cells are in their environment (e.g., via stress-induced mutagenesis [41]), we choose to fix *k* to a constant value here for expositional purposes.

Naturally, cells may get trapped on local peaks (shown by the purple stars) and never reach the global peak (shown by the red star) of the adaptive landscape. Getting out of local peaks requires either the adaptive landscape to change in such a way that it no longer remains a peak or for the species to produce enough genetic variation to step over the valley [18]. In cancer, an example of this can be seen when nutrient composition in the environment changes drastically. Though an entire rewiring of metabolic circuitry could lead to a global optimum with respect to resource utilization, this is often evolutionarily unfeasible as it requires cells to pass over several valleys in the process of modifying their metabolic networks. On the other hand, a slight shift in the expression levels of proteins implicated in metabolism may be enough to reach a local optimum—this is the strategy that will be evolutionarily preferred.

Table 1 summarizes the important components so far in the population and strategy dynamics.

**Table 1:** Key parameters in population and strategy dynamics

Parameter	Interpretation
*G*	Per capita growth rate of a clone
*v*	Strategy of focal cell
${\textbf{u}}$	Strategies of each clone in population
${\textbf{x}}$	Population size of each clone in population
*k*	Trait evolvability
$\frac{dG}{dv}$	Gradient of *G* function

## Results

### Basic cancer growth

Now that we have outlined the core ecological and evolutionary dynamics, we are ready to build *G* functions in the context of cancer. To do this, we follow the following three steps: (1) create a model of the ecological dynamics, (2) identify relevant traits that can influence these dynamics, and (3) outline how the focal cell’s strategy (*v*) and the strategies of other clones in the population (**u**) affect values of key parameters in our model.

To model the ecological dynamics of cancer cells (i.e. how the number of cancer cells in the population changes over time), we must first consider their growth rate. We may start by assuming that the per capita growth rate (*G*) is constant, independent of population size. Under this assumption, $G=r,$ where *r* is the intrinsic growth rate (division rate-death rate per time step) and thus $\frac{\mathrm{{d}}x}{\mathrm{{d}}t} = rx$. This is a model of exponential growth: assuming $r>0$ (division rate exceeds death rate), the population will increase exponentially without bound. Exponential growth is a good description for the initial phase of growth for a population under optimal conditions, in which resources and space are plentiful [60]. However, it is not a good predictor of the long-term dynamics of cancer cells. Namely, the exponential growth model does not consider density-dependent factors such as nutrient depletion. which make the per capita growth rate decrease with population density rather than remain constant.

One way we can take this into account is to let the per capita growth rate decrease linearly with population size. Mathematically, this is represented by $G=r(1-x/K)$. Here, *r* is the intrinsic rate of increase: the maximum growth rate when the population size is small. Clinically and experimentally, this can be calculated from the doubling time of a cell population with ample space and nutrients as *ln*(2)/*D* where *D* is the doubling time. *K* is the carrying capacity, the maximum number of cells the environment can support. In the lab, this can be detected from population counts when cells reach confluence and growth has stagnated. Below this level, the overall per capita growth rate is positive, at the carrying capacity no growth occurs, and above it the growth rate is negative. Thus, our basic ecological dynamics can be written as follows:

3 $$\begin{aligned} \frac{\mathrm{{d}}x}{\mathrm{{d}}t} = rx\left( 1-\frac{x}{K}\right) . \end{aligned}$$

The notion of a carrying capacity is widely debated in the literature–after all, cancer is characterized by an unlimited potential for growth [29, 48, 52, 81, 101, 105]. Despite this, it is widely accepted that competition among cells for limited resources leads to a carrying capacity. Although this carrying capacity may be incredibly high, there are only so many cancer cells a human can host before succumbing to the disease. It is also worth noting that, while there are several other growth models used in modeling cancer such as Gompertz and Bertalanffy growth [72, 94, 107], we choose to use logistic growth for expositional purposes: this is the most widely used growth model in the mathematical oncology literature.

Now, we must decide what the relevant evolutionary traits are that are associated with cellular dynamics. Since we are focused on basic cancer growth here, we let our strategy represent a “growth strategy” for the cancer cells. Intentionally kept general, this could represent transporter production for nutrient uptake or the use of certain metabolic pathways for cell growth and division. Next, we must determine which parameters in our model will depend on this strategy. In order to see the clearest impact on population and strategy dynamics, we choose to let the carrying capacity, *K*, depend on *v*. Namely, we assume that a strategy of $v=0$ maximizes a clone’s carrying capacity. Deviations from this decrease the carrying capacity in a Gaussian fashion, as described below. For instance, over- or under-producing nutrient transporters can lead to suboptimal growth at the clonal level.

4 $$\begin{aligned} K(v) = K_mexp\left[ -\frac{v^2}{2\sigma _k^2}\right] . \end{aligned}$$

**Fig. 2:**
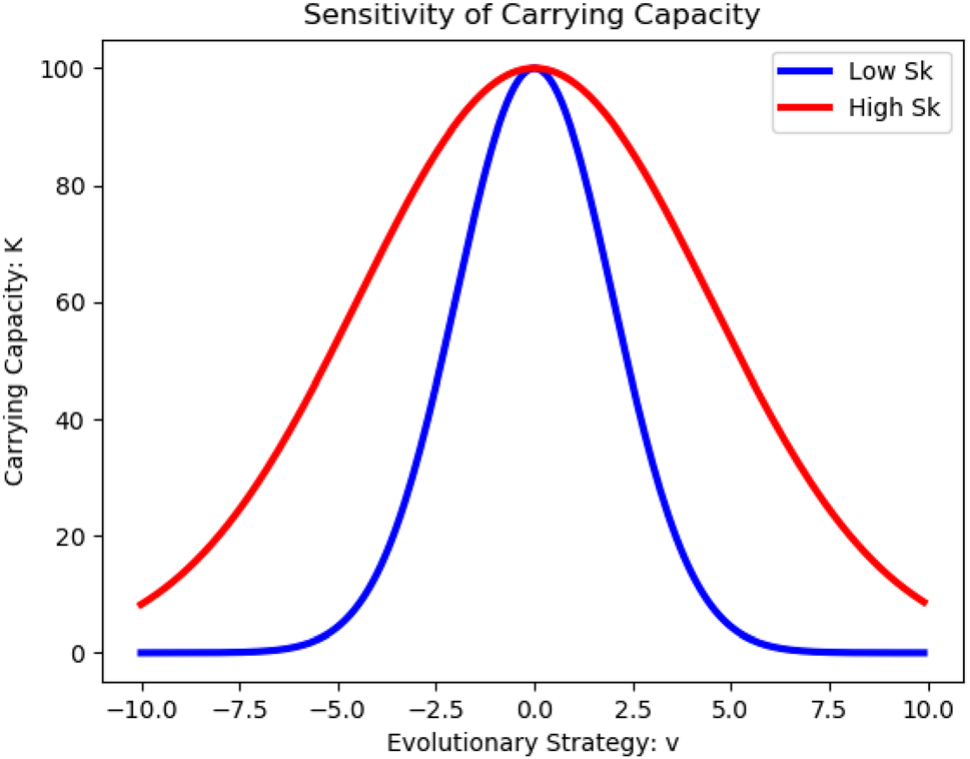
Sensitivity of Carrying Capacity to Changes in $\sigma _k^2$. A sample carrying capacity function is plotted here with $K_m=100$. The blue curve depicts a carrying capacity with a high sensitivity to changes in *v* with $\sigma _k^2=4$. The red curve represents a carrying capacity that is relatively insensitive to changes in *v* with $\sigma _k^2=20$. As we can see, the carrying capacity of the blue curve drops off sharply if the strategy deviates even a little from 0. In comparison, deviances from $v=0$ impact the red curve’s carrying capacity less

Here, $K_m$ denotes the absolute carrying capacity, achieved at $v=0$. The breadth of the Gaussian distribution is modulated by $\sigma _k^2$. The smaller the value of $\sigma _k^2$ is, the more sensitive the carrying capacity is to changes in *v*. In other words, if small perturbations from the optimal value of the relevant trait only modestly change the number of cells at confluence, $\sigma _k^2$ will be small. However, if minor perturbations have a major impact on carrying capacity $\sigma _k^2$ will be large. This idea is illustrated in Fig. 2.

Although this formulation of carrying capacity strictly only holds in the monomorphic case (as we’re dealing with here), it can easily be extended to the case of a polymorphic population, either by allowing each clone to retain its own carrying capacity or by enforcing a population-level carrying capacity as the weighted average of the carrying capacities of all clones. Our *G* function can now be written as

5 $$\begin{aligned} G(v,{\textbf {x}}) = r\left( 1-\frac{x}{K(v)}\right) . \end{aligned}$$

Plugging this in for *G* in Eqs. 1 and 2, we derive our population and strategy dynamics to be:

6a $$\begin{aligned} \frac{\mathrm{{d}}x}{\mathrm{{d}}t} = rx\left( 1-\frac{x}{K(v)}\right) , \end{aligned}$$

6b $$\begin{aligned} \frac{\mathrm{{d}}u}{\mathrm{{d}}t} = k\frac{-rux*exp\left( \frac{u^2}{2\sigma _k^2}\right) }{K_m\sigma _k^2}. \end{aligned}$$

**Fig. 3:**
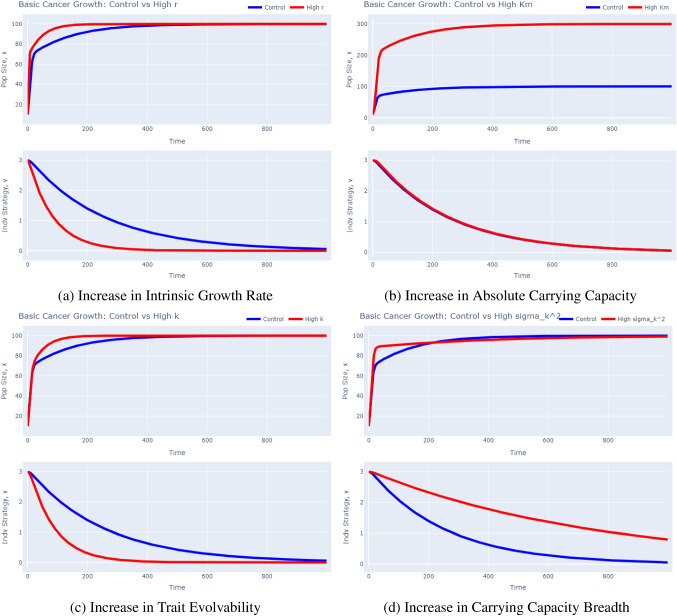
Basic Cancer Growth Simulations: Impact of Increases in $r,K_m,k,$ or $\sigma _k^2$ on Population and Strategy Dynamics. These plots were produced using the *G*-function software tool’s “Basic Growth” model. To recreate these plots, one must change the respective “modified species” parameter value in the advanced drop down menu to thrice the value of the “control species”. For example, to generate the “Control vs High r” plot, set $r_2$ to 0.75 and run the model

We are now ready to simulate the eco-evolutionary dynamics of our cancer cells. To build an intuition for our model, we vary each parameter value and see how it affects cancer cell dynamics when compared to a control simulation in Fig. 3. The base parameter values used in the control simulation are given in Table 2 and are the default values used in the *G*-function software tool.

**Table 2:** Control simulation parameter values

Parameter	Interpretation	Value
$\mathbf {x_0}$	Initial population size of each clone	10
$\mathbf {v_0}$	Initial strategy value of each clone	3
$K_m$	Absolute carrying capacity	100
*r*	Intrinsic growth rate	0.25
$\sigma _k^2$	Breadth of carrying capacity	12.5
*k*	Trait evolvability	0.2

In each of the panels in Fig. 3, the top graph represents population dynamics and the bottom graph represents strategy dynamics. First, we make a few general observations. In all cases, the cancer cells gradually evolve towards a strategy of $v=0$ to maximize their carrying capacity. Considering ecological dynamics, we see a sharp increase in population size at the beginning followed by a more gradual increase, characteristic of logistic growth. The population size tapers off at the absolute carrying capacity at $x=100$ at the end of the simulation. Now, let us specifically look at the impact of an increase in each parameter value (tripling of the control value) on the eco-evolutionary dynamics of cancer cells.

First, consider Fig. 3a in which we simulate a high intrinsic growth rate. In this case, note that the strategy evolves much more quickly to the equilibrium at $v=0$. As we can see from Eq. 6b, this is because *r* directly increases the speed of evolution at each time step. We also see that the population reaches the absolute carrying capacity at $x=100$ much more quickly. The reason behind this is two-fold. Considering Eq. 6a, the two main parameters which govern population increase are *r* and *K*(*v*). By default, a higher *r* results in a faster cell division rate, aiding population growth. But it also increases *K*(*v*) at a faster rate since *v* reaches its optimal value at 0 more quickly.

Next, we turn our attention to Fig. 3b that depicts a high absolute carrying capacity. As one would expect, the cancer cell population now reaches a carrying capacity of 300 instead of one at 100. The initial population burst is more notable, owing to the higher carrying capacity that does not constrain growth at low population sizes as strongly. Furthermore, note that the strategy dynamics for the control and high carrying capacity case are nearly identical. This is because the higher $K_m$ is offset by a higher *x* in Eq. 6b.

Now, consider Fig. 3c, in which we simulated a higher evolvability of the trait. As expected from Eq. 6b, the strategy now evolves much more quickly to an equilibrium of $v=0$ since the “step size” of evolution at each time step is now increased. We also notice that the population reaches its absolute carrying capacity more quickly. Since the trait evolves to more optimal levels more quickly, *K*(*v*) increases more rapidly, restraining population growth to a lesser degree.

Finally, consider Fig. 3d where we made the carrying capacity less sensitive to trait values that diverge from $v=0$. The selection pressure for the trait to evolve to 0 is now reduced and the adaptive landscape will correspondingly be less steep. This leads to a slower evolution of the trait value. There are two things to note in the population dynamics. First, observe the more rapid growth for a less sensitive carrying capacity at the beginning. This is because *K*(*v*) remains relatively high the entire time and thus does not constrain population growth as much, *initially*. However, we also see that it takes longer to reach the absolute carrying capacity. This is because the trait evolves more slowly to 0, leaving the cancer cells off-peak for a longer period of time.

To summarize, we have created a simple model of cancer growth and found (1) a higher intrinsic growth rate not only increases population size more quickly, but also speeds up the rate of evolution, (2) a higher carrying capacity allows for a greater number of cells to exist in the population, leading to a higher population size at equilibrium, (3) a higher evolvability causes a faster rate of evolution and allows the population to reach carrying capacity sooner, and (4) a carrying capacity that is less sensitive to trait value decreases the selection gradient by allowing cells with trait values far from $v=0$ to proliferate effectively: this leads to a higher *initial* population growth but a slower rate of trait evolution.

### Cancer cell competition

One assumption implicit in our model is that all cells compete equally with each other. However, this may not always be realistic. For example, cells that use similar metabolic pathways may experience greater competition with each other for nutrients than with cells that have different nutrient requirements. To incorporate this intraspecific competition into our model, we can amend our *G* function from Eq. 7 as follows:

7 $$\begin{aligned} G(v,{\textbf {u}},{\textbf {x}}) = r\left( 1-\frac{\sum _{i}a(v,u_i)x_i}{K(v)}\right), \end{aligned}$$

where the intraspecific competition function, $a(v,u_i)$, scales how each clone diminishes the per capita growth rate of another depending on the strategies of the focal and competing clones. These parameter values can be experimentally informed via a series of competition assays. Note that this function can be asymmetric: if clone 1 outcompetes clone 2 when cultured together, we can assume that $a(u_1,u_2)<a(u_2,u_1)$, i.e., clone 1 negatively impacts clone 2’s growth more than clone 2 impacts clone 1’s growth. We reintroduce the subscripts on *x* and *u* to make explicit the fact that we are dealing with more than one clone in our population. Here, we assume that like competes most with like:

8 $$\begin{aligned} a(v,u_i) = exp\left[ -\frac{(v-u_i)^2}{2\sigma _a^2}\right] . \end{aligned}$$

Under this framework, cells of two clones will compete more if their trait values are similar rather than dissimilar. This competition function has the property that when a focal cell has the same trait value as that of its competitor, $a(v,u_i) = 1$. The $\sigma _a^2$ term represents the breadth of the competition function. The smaller the value of $\sigma _a$, the more effectively cells of different clones can avoid competition from one another by having divergent trait values and thus occupying different niches.

**Fig. 4:**
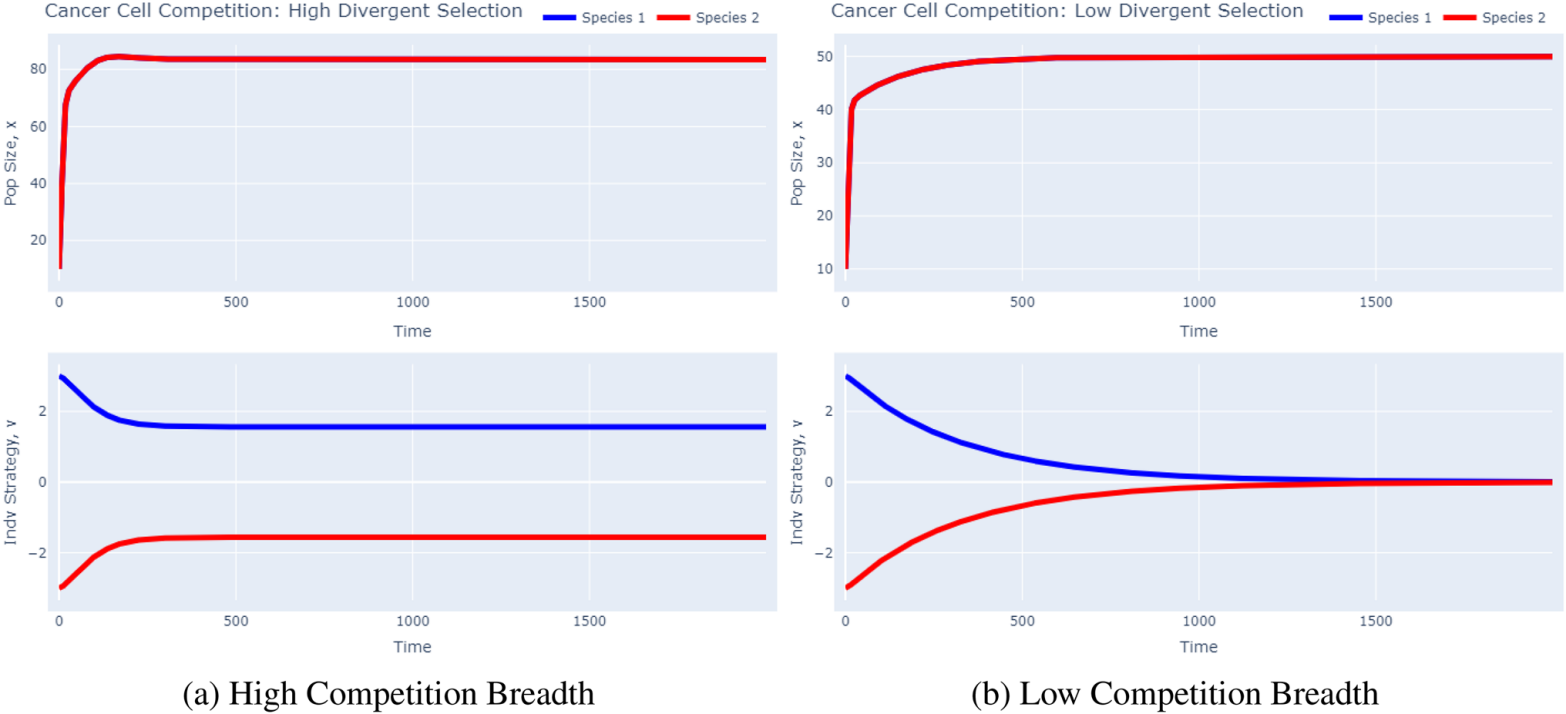
Cancer Growth with Competition Dynamics. Impact of $\sigma _a^2$: Breadth of Competition Function. These plots were produced using the *G*-function software tool’s “Competition” model. To recreate the left plot, simply run the model as is. To recreate the right plot, change the value of $\sigma _a$ in the advanced drop down menu to 50 and run the model

Now, we run simulations in Fig. 4 with two competing clones, distinguished only by their initial strategy. We let one clone start with $v(0)=3$ and the other with $v(0)=-3$. We use the same parameters as given in Table 1 and run two simulations: one with $\sigma _a^2=2$, corresponding to clones that can effectively avoid competition from one another by having divergent trait values, and one with $\sigma _a^2=50$, capturing a case in which trait value divergence does not help much in avoiding competition.

In this case, there is a trade-off between maximizing carrying capacity and avoiding competition. When the breadth of the competition function is low, as in Fig. 4a, clones are effectively able to avoid competition with each other. This can be seen by the two clones having different strategy equilibria at $\approx$ 1.56 and $-$1.56. Note that the clones do not reach their absolute carrying capacity at 100 since they still remain far from the strategy which maximizes carrying capacity ($v=0$). Now, consider Fig. 4b in which the breadth of the competition function is high. Here, having a divergent trait value does not help clones much in avoiding competition from one another. Thus, the benefit of maximizing carrying capacity outweighs the minimal benefit the clones may gain from having divergent strategies. This leads to a convergence in strategy values at $v=0$. Note that the population size of the clones is even less in this case, each at 50. This is because, even though the clones are at $v=0$, they are experiencing maximal competition from one another. In other words, they are equally inhibited by their own growth as they are from their competitors. This is what leads them to each occupy half of the absolute carrying capacity of each–they are both functionally equal competitors coexisting in the same niche with a carrying capacity of 100.

Note that in these simulations, we assumed a small niche breadth. This promoted coexistence of clones as they were effectively able to avoid competition from one another. However, if this niche were to be expanded, we would notice competitive exclusion: the clone that starts closer to the evolutionary equilibrium would outcompete the other.

### Drug resistance

Now, we consider a problem of clinical relevance: the emergence of drug resistance after the administration of therapy. First, we consider the ecological component of our problem. Although cancer is an incredibly complex disease involving intricate interactions among heterogeneous cancer cells, normal cells, immune cells, etc., we choose to just focus on cancer cell dynamics here. We start with logistic growth (Eq. 3) as our base and add on the effects of therapy. Specifically, we assume cancer cells die in a density-independent fashion (i.e. constant *per capita* death rate) due to therapy. Thus, our ecological dynamics are:

9 $$\begin{aligned} \frac{\mathrm{{d}}x}{\mathrm{{d}}t} = rx\left( 1-\frac{x}{K}\right) -sx, \end{aligned}$$

where *x* is the population of cancer cells, *r* is the intrinsic growth rate of the cancer cells, *K* is the carrying capacity, and *s* is the drug’s killing efficiency. Now, we must define the key strategies associated with cancer cell dynamics. Since we are trying to keep this model as general as possible, the exact meaning of our key strategy varies from treatment to treatment. In the context of glucose starvation, for example, it may represent an increase/decrease in GLUT1 transporters or in the context of a BRAF inhibitor, it may quantify changes in the intracellular MAPK pathway. Now, we must determine which parameters in the model will vary based on *v*, **u**, and **x**. We let the carrying capacity and killing rate vary as a function of the focal strategy. Namely, we assume that when $v=0$, the clone maximizes its carrying capacity. Any deviation from this causes the carrying capacity to decline according to a Gaussian distribution:

10 $$\begin{aligned} K(v) = K_mexp\left[ -\frac{v^2}{2\sigma _k^2}\right] . \end{aligned}$$

Here, $K_m$ denotes the absolute carrying capacity and $\sigma _k^2$ modulates the breadth of the Gaussian distribution (see Fig. 2). We use a similar form for the drug’s killing efficacy:

11 $$\begin{aligned} s(v) = s_mexp\left[ -\frac{(v-u_{opt})^2}{2\sigma _t^2}\right] . \end{aligned}$$

Here, $s_m$ is the maximal killing rate of the drug, $u_{opt}$ is the cancer cell strategy at which the drug is most effective, and $\sigma _t^2$ is a measure of the generality of the drug: how effective the drug is when cancer cells deviate from $u_{opt }$. These parameters can be informed by dose-response curves that measure the death due to drug over time. We now have a fully constructed *G* function model. Note that, since the *G* function gives the *per capita* growth rate of the clone, we have:

12 $$\begin{aligned} G(v,{\textbf {u}},{\textbf {x}}) = r\left[ 1-\frac{x}{K(v)}\right] -s(v). \end{aligned}$$

**Fig. 5:**
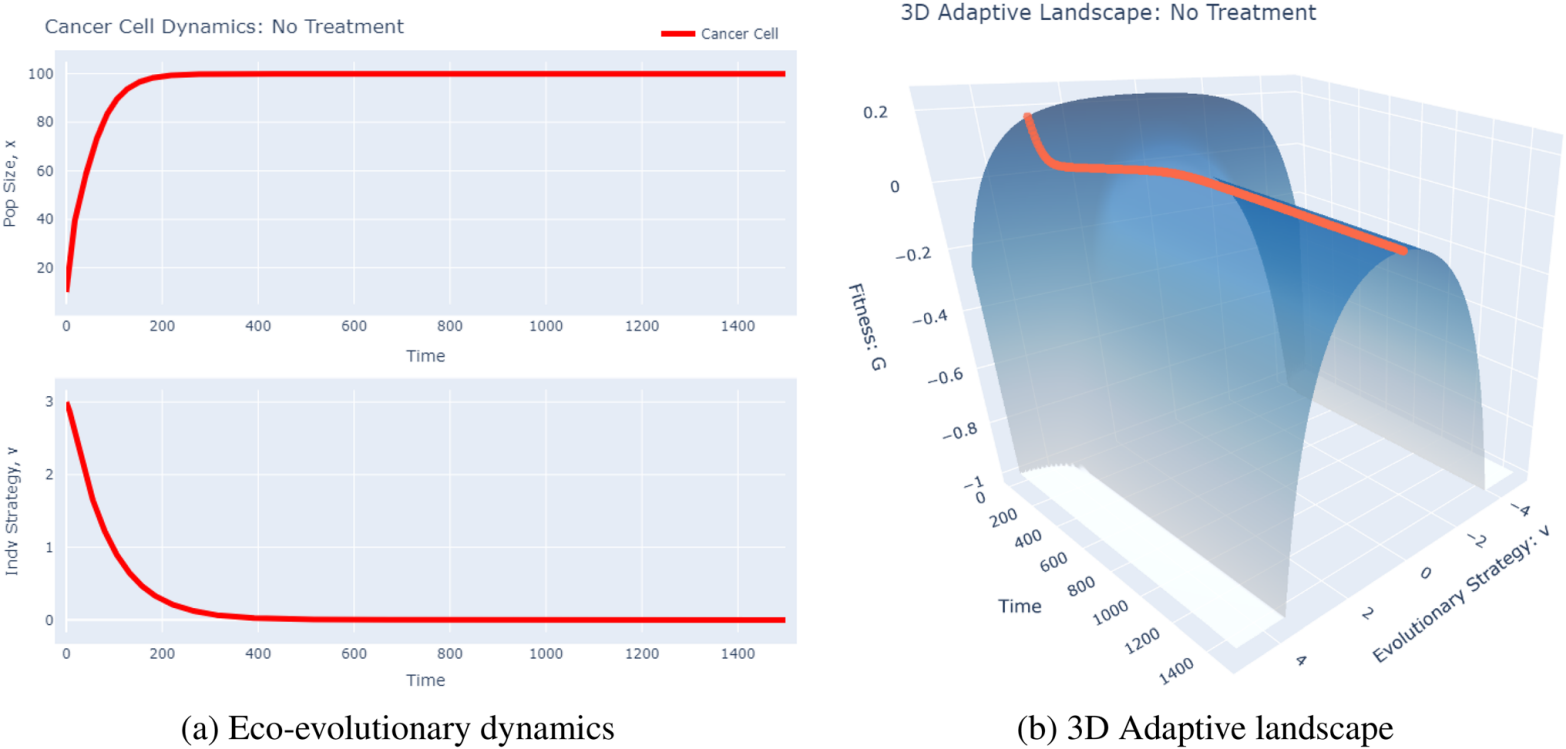
No Treatment Simulations. These plots were produced using the *G*-function software tool’s “Drug Resistance” model. To recreate these plots, simply run the model as is using the 2D graph type (left panel) and 3D graph type (right panel)

We can now run some simulations to examine what happens to the population and strategy dynamics of the cancer cells over time. First, we consider the non-treatment control case. We start the cancer cell population at $x(0)=10$ and the strategy at $v(0)=3$ and simulate the model for 1500 time steps. In addition to plots of the population and strategy dynamics, we create a 3D plot of the evolution of the adaptive landscape over time (Fig. 5).

**Fig. 6:**
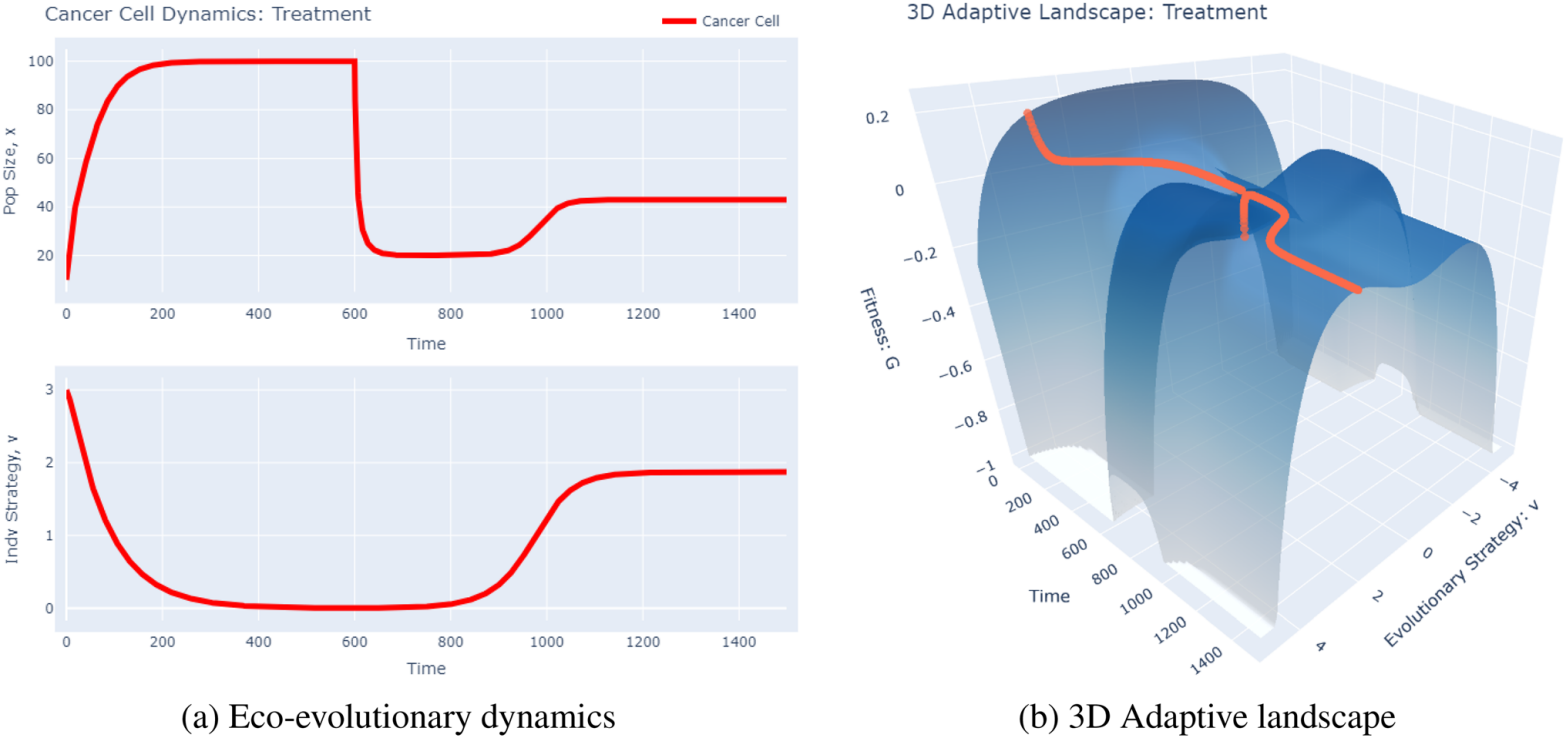
Treatment Simulations. These plots were produced using the *G*-function software tool’s “Drug Resistance” model. To recreate these plots, change the “Time to Start of Treatment (time_G)” parameter to 600 and run the model using the 2D graph type (left panel) and 3D graph type (right panel)

There are a few things to notice here. First, as we would expect, the population evolves towards $v=0$, the strategy at which their carrying capacity is maximized. We see that the population does reach their maximal carrying capacity at 100 in a logistic fashion. When we consider the adaptive landscape, we see that, for the entirety of the simulation, there is only one peak in the population, corresponding to $v=0$; the fitness of the cancer cells drops off as the cells deviate from this strategy. Now, let us turn our attention to the treatment case (Fig. 6). Here, using the same initial conditions as above, we induce a treatment (maximally effective for cancer cells with $v=0$) at time 600.

We notice very different dynamics in this case. Just before treatment is applied, the population has reached its ecological and evolutionary equilibria at $x=100$ and $v=0$. The adaptive landscape at this time is identical to that of the no treatment case. After administration of the drug, we notice the population density initially decreases sharply. From the adaptive landscape, we can see that this occurs because the $v=0$ strategy now corresponds to a valley in the adaptive landscape. In response to treatment, the population evolves a new strategy by climbing the adaptive landscape to a new peak, resulting in an increase in size. Notice that there are two peaks of equal fitness in the adaptive landscape, implying the potential for speciation to occur with cells evolving two different strategies to evade the effects of treatment. In the case of BRAF inhibitor resistance in melanoma, for example, this speciation event may occur through activation of RTKs like IGF-1R or through engagement of the PI3K pathway via PTEN inactivation [62, 102]. This sharp change in the adaptive landscape from a unimodal to bimodal distribution can clearly be seen in the adaptive landscape.

The new strategy at $v \approx 2$ is a balance between two opposing selective forces. On one side is the stabilizing selection exerted by maximizing carrying capacity, which tries to pull the population as close as possible to $v=0$. On the other side is the divergent selection exerted by treatment, which pushes the population as far away as possible from $v=0$. Note, however, that the post-treatment population equilibrium is much lower than the pre-treatment equilibrium. Intuitively, this is because, although cancer cells may use different intracellular signaling pathways to evade the effects of BRAF inhibitors, these alternative pathways are inherently less energetically efficient, leading to a lower overall fitness and growth rate of the population. Although we implement a simple therapeutic protocol here, more intricate evolutionarily informed dosing strategies can be explored by expanding the model to include several therapies and therapy periods. We refer the readers to our prior work to see how this is done [18, 103].

To summarize, our model demonstrates the ecological and evolutionary underpinnings of drug resistance. Although alternate strategies (e.g., metabolic pathways) may be suboptimal in the absence of therapy, the administration of an appropriate drug creates a valley at the prior optimal strategy. This valley leads to new peaks that may allow for speciation of the population. Therefore, therapy may be a source of diversification and heterogeneity. Although we have presented a simplified version of the evolution of drug resistance in cancer, there are many ways this model can be extended, depending on the question of interest. For example, one could consider the influence of other cell types such as immune cells and fibroblasts, spatiotemporal variation and heterogeneity in the microenvironment [27, 61, 85], side effects of drugs [86], drug scheduling [109], evolutionarily informed therapies [18, 19], and the effects of plasticity and cell states [20, 21].

## Discussion

Cancer research has made much progress over the last several centuries, from understanding the functions of key genes and proteins and outlining detailed molecular mechanisms that promote tumorigenesis to the advent of therapies such as radiation therapy, chemotherapy, and immunotherapy. However, patient outcomes are still dismal in many regards. To truly understand and effectively treat cancer, we must supplement the gene-centric paradigm that dominates much of modern cancer research with a higher-level understanding of the ecological and evolutionary forces that shape cancer.

It has long been recognized that cancer cells are groups of heterogeneous cells that are evolving in a constantly changing ecological environment, subject to fluctuations in nutrient and oxygen levels, “predation” by immune cells, and therapy [7, 78]. Ecological and evolutionary perspectives are necessary to understand the complex nature of cancer biology and develop more effective treatment strategies. Much work in last several decades has applied ecological and evolutionary theory to a diverse set of problems in cancer biology [100] including the origins of cancer [5, 34, 79, 80, 84], tumor-microenvironment coevolution [13, 67, 73, 77, 99], cell signaling [17], infection and cancer [37–39], metastasis [8, 9, 24, 40, 50, 54, 82, 93], dormancy [4, 71, 98], and of course therapeutic responses [6, 11, 16, 27, 36, 42, 43, 46, 70]. In fact, many of the earliest mathematical models of cancer such as the logistic, von Bertalanffy, and Gompertz growth models were inspired by problems in ecology. More recently, these evolutionary perspectives into tumorigenesis were formalized through the use of EGT [106], starting with the pioneering work of Tomlinson who used matrix games to probe competition among cancer cells [97]. Since then, the scope of EGT as a tool to solve problems in cancer has expanded greatly, due to both mathematical advancements in EGT [23] and greater awareness of the usefulness of such techniques to solve biological problems [18]. Current approaches now allow for incorporation of spatial heterogeneity [1, 10, 14, 25, 56, 63, 75, 96, 106, 108], optimization of cancer treatment [22, 26, 49, 53, 64, 90, 104], linking game theoretic frameworks to experimental [55, 92] and clinical patient [65, 86, 88] data to drive and inform clinical trials.

However, a simple, unifying modeling framework that allows us to simultaneously consider the ecological and evolutionary dynamics of cancer in a variety of scenarios is lacking. In this paper, we show how the *G*-function modeling framework can help us do just this. Starting from first principles, we walk through the development of *G* functions and show its power to simultaneously model ecological (population) and evolutionary (strategy) dynamics. We then use *G*-functions to construct a basic model of cancer growth, upon which we add cancer cell-cell competition and analyze a basic scenario of the evolution of therapeutic resistance. Overall, the *G* function framework allows us to rigorously create simple mathematical models of our hypotheses about the natural world and provides the necessary tools to simulate and analyze the resulting models. These results can then be compared to reality and, if needed, the hypotheses modified in an iterative fashion.

## Data Availability

All model simulation plots were produced from the *G*-function software tool, found at https://lively-wave-033dd4510.azurestaticapps.net/. The codes behind the software tool can be found at https://github.com/ihockett01/g-function-api
